# Identification of Proteins Modulated in the Date Palm Stem Infested with Red Palm Weevil (*Rhynchophorus ferrugineus* Oliv.) Using Two Dimensional Differential Gel Electrophoresis and Mass Spectrometry

**DOI:** 10.3390/ijms160819326

**Published:** 2015-08-17

**Authors:** Khawaja Ghulam Rasool, Muhammad Altaf Khan, Abdulrahman Saad Aldawood, Muhammad Tufail, Muhammad Mukhtar, Makio Takeda

**Affiliations:** 1 Department of Plant Protection, College of Food and Agriculture Sciences, King Saud University, Riyadh 11451, Saudi Arabia; E-Mails: aldawood@ksu.edu.sa (A.S.A.); mtufail@ksu.edu.sa (M.T.); 2 Graduate School of Agricultural Science, Kobe University, Kobe 657-8501, Japan; E-Mail: mtakeda@kobe-u.ac.jp; 3 Department of Plant Production, College of Food and Agriculture Sciences, King Saud University, Riyadh 11451, Saudi Arabia; E-Mail: altafksu@gmail.com; 4 Department of Biotechnology, American University of Ras Al Khaimah, Ras Al Khaimah 10021, United Arab Emirates

**Keywords:** date palm, stem, red palm weevil, infestation, differential expression, proteins, two dimensional differential in-gel electrophoresis (2D-DIGE), Matrix Assisted Laser Desorption/Ionization-Time of Flight (MALDI TOF)

## Abstract

A state of the art proteomic methodology using Matrix Assisted Laser Desorption/Ionization-Time of Flight (MALDI TOF) has been employed to characterize peptides modulated in the date palm stem subsequent to infestation with red palm weevil (RPW). Our analyses revealed 32 differentially expressed peptides associated with RPW infestation in date palm stem. To identify RPW infestation associated peptides (I), artificially wounded plants (W) were used as additional control beside uninfested plants, a conventional control (C). A constant unique pattern of differential expression in infested (I), wounded (W) stem samples compared to control (C) was observed. The upregulated proteins showed relative fold intensity in order of I > W and downregulated spots trend as W > I, a quite interesting pattern. This study also reveals that artificially wounding of date palm stem affects almost the same proteins as infestation; however, relative intensity is quite lower than in infested samples both in up and downregulated spots. All 32 differentially expressed spots were subjected to MALDI-TOF analysis for their identification and we were able to match 21 proteins in the already existing databases. Relatively significant modulated expression pattern of a number of peptides in infested plants predicts the possibility of developing a quick and reliable molecular methodology for detecting plants infested with date palm.

## 1. Introduction

The red palm weevil (RPW) (*Rhynchophorus ferrugineus* Oliv., Coleoptera: Curculionidae) has become the most destructive pest of date palm trees in several regions of the world including Saudi Arabia. This palm-damaging pest was first reported in Southeast Asia on coconut palm [1]. Since its discovery in the Gulf Region in the year 1980s, the insect has been spreading rapidly and reported from almost every palm growing country in the World [2]. Bulk movement of date palm offshoots for planting is blamed on the invasion source of RPW in the Middle East [3]. The RPW has been reported to infest 26 palm species belonging to 16 different genera worldwide [4]. Although it is difficult to evaluate the overall actual global damages caused by RPW, in Saudi Arabia along with just ~5% infestation; management and eradication of RPW in date plantation cause more than 8.69 million USD of economic loss [5].

It is also worth mentioning that RPW larval stage is the most destructive, and responsible for damaging the palm. The larvae feed within the date palm trunk until they are fully developed [6,7]. This insect completes several generations within the same palm without any obvious symptoms in the plant until the tree finally collapses [8,9]. This cryptic feeding behavior of the RPW makes it difficult to detect infestations at early stages, and severe decaying of the internal tissues leads to the death of the tree [3,10].

In Saudi Arabia, the Ministry of Agriculture has launched a national campaign for controlling RPW to avoid losses inflicted on the production of dates. The campaign includes removal of infested plants, pesticide application through injection and spraying in severely infested and newly infested areas, and the use of pheromone traps for monitoring and decreasing RPW populations [11]. It has been observed that infested plants can be recovered if infestation is detected early. Currently available detection techniques, including visual inspections, acoustic sensors [12,13], sniffer dogs [14], and pheromone traps [15], are in practice to identify infestations at early stages. However, the development of an effective and efficient high throughput screening procedure is still needed for the early detection of RPW.

We employed proteomic methodologies to identify responses associated with RPW infestation. It has been previously reported that plants have evolved various innate and acquired defense mechanisms against visible/invisible injuries afflicted by insect pests [16]. Innate or direct defense mechanisms in plants include specialized characteristics, such as thorns, trichomes, and primary and secondary metabolites [16]. Some herbivores feeding induce proteinase inhibitors in plants that prevent digestive enzymes required for insect’s proper digestion thus limiting invasion [17]. Acquired defenses involve release of volatile organic compounds that attract arthropod predators and parasitoids to control herbivore populations [18]. Herbivores oral secretions specially discharged into plant tissues during feeding induce a number of specific responses [19,20]. The herbivores regurgitate and other oral secretions trigger plant defense related proteins (parallel to acquired immune system of mammals) or activate the plant defense system releasing volatile compounds to attract predators [21,22].

Studies also confirm differential molecular defensive responses upon infestation with a wide variety of insects/pests. As the plant genomics/proteomics related to plant defense responses is comparatively a new field of study, more data is emerging to better understand plant responses against biotic and abiotic stresses [23]. The insect plant interactions itself have great impact on plant defense responses [16,24].

Proteomics strategies have been extensively used for identifying infections/diseases among humans; however their uses for plants have been relatively less. A few proteomic studies involving plants encouraged us to embark on utilizing these methodologies for saving beneficial date palm plants from RPW infestation. For example, proteome analysis of brittle leaf diseased date palm leaves when compared with that of their normal counterparts revealed quantitative differences in several proteins. Of the differentially expressed proteins, Manganese (Mn-binding) PSBO and PSBP proteins were downregulated; whereas, several other proteins were upregulated in diseased samples [25]. Likewise, proteome analysis of brittle leaf disease affected date palm leaves indicated changes in the proteome at early disease stage where the decrease in Mn deficiency associated with MSP-33 kDa subunit protein was considered as brittle leaf disease biomarker [26]. Moreover, Gómez-Vidal *et al.* [27] have evaluated the plant defense/stress, photosynthesis and energy metabolism related proteins, using 2-dimensional electrophoresis (2DE) proteomic techniques, that were differentially expressed in the date palm (*Phoenix dactylifera*) leaves in response to the attack by entomopathogenic fungi (*Beauveria bassiana, Lecanicillium dimorphum* and *L. cf.* *psalliotae*) as compared to control samples. In another study, 2DE of date palm sap 52 identified spots among 100 were related to *Saccharomyces cerevisiae*, supposed to be natural microflora of date palm sap while others were related to vegetable proteins playing a role in the vascular system of the plant [28]. Several changes in date palm leaf proteome were observed when red palm weevil infested and healthy date palm leaves proteome were compared [29,30]. Furthermore, we have a highly integrated proteomic initiative to identify molecular markers associated with RPW infestation and other diseases to this plant. As far as the proteome defenses between leaf and stem are concerned, we observed different protein moieties modulated in stem and leaves. However, an extensive analysis regarding this aspect is ongoing. Modulations in the date palm fruit proteome have also been reported during development and ripening stages [31]. In another study two dimensional differential gel electrophoresis and mass spectrometry also revealed changes in the proteome of salinity and drought-stressed palm seedlings when compared with non-stressed plants [32].

Very few studies have addressed the date palm proteomics association with physiological or induced changes in date palm. Our study is unique in a way that it provides proteomic changes associated with RPW infestation and its comparison with artificially induced injury to plant.

As such, the objective of the present study was to characterize the proteome changes occurring in date palm stem infested with RPW using 2D-DIGE and mass spectrometry so as to explore the biomarker for the early detection of RPW for its effective management.

## 2. Results and Discussion

### 2.1. Date Palm Proteome Analysis by 2D-DIGE

**Figure 1:**
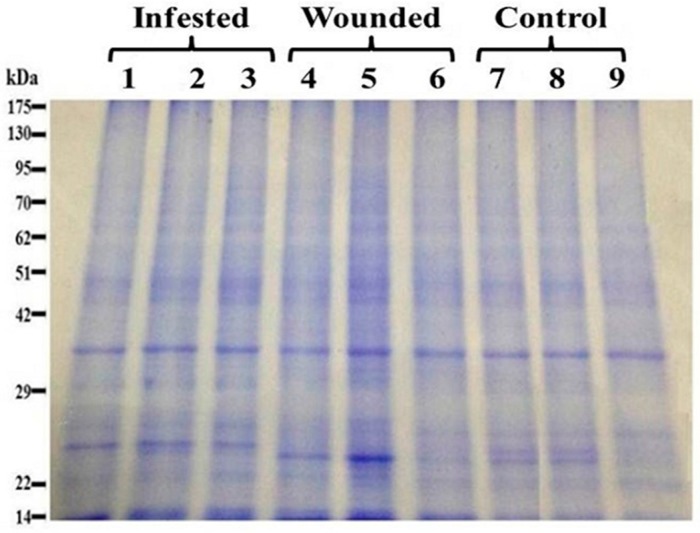
Comparative protein expression profiling of the control, infested and wounded date palm samples using SDS-PAGE. Lanes 1–3 represent total cell proteins from 3-infested replicates; while lanes 4–6 represent proteins from wounded date palm samples; and lanes 7–9 represent proteins from control date palm samples.

Plants respond to injuries/infestations and other abiotic stresses by activating a broad range of acquired defense system, including activation of pathogenesis-related (PR) genes both at local and systemic sites [16], crosslinking of cell wall proteins, generation of reactive oxygen species (ROS), and local programmed cell death. This study reports our ongoing efforts to understand proteomic modulation associated with RPW infestation. Previously, we optimized protein isolation from date palm leaves and its utilization in proteomic evaluations. This study extrapolates our work on the stem part of the date palm plants. As expected, different peptides were modulated in stem as the metabolic activities of leaves are quite different. We identified eleven peptides modulated in the leaves of date palm belonging to three main categories *i.e.*, stress/defense, photosynthesis, and ion transport [29]. For evaluating differential proteomic responses subsequent to infestation stem samples from infested, control, and artificially wounded plants were subjected to protein isolation followed by differential expression analyses. Proteins were extracted from stem samples using phenol- sodium dodecyl sulfate (SDS) extraction method [33]. For reducing internal variations three replicates for each plant were used. The extracted proteins were then quantified using 2D quant kit after solubilizing in 2D-rehyration buffer. Ten µg aliquots of each sample was solubilized in SDS loading buffer and separated on 12.5% sodium dodecyl sulfate polyacrylamide gel electrophoresis (SDS-PAGE) before staining. Protein profile after staining with Coomassie showed good reproducibility among replicates, consistent solubilization and reproducible extraction methods (Figure 1).

Moreover, 2D DIGE (General Electric (GE) Healthcare, Buckinghamshire, United Kingdom) was run to compare differences among control, infested, and wounded samples. These samples were labeled with either Cy3 or Cy5 dyes while the internal standard was consistently labeled with Cy2. The experimental design for 2D-DIGE experiments is shown in (Table 1).

**Table 1:** Experimental design for 2D-DIGE. Three replicates from each control, infested, and wounded protein samples were labeled and combined for 2D-DIGE.

Gel Number	Protein Samples Labeling		
	Cy2	Cy3	Cy5
1	Pooled sample	Infested R1	Control R3
2	Pooled sample	Infested R2	Wounded R1
3	Pooled sample	Infested R3	Wounded R2
4	Pooled sample	Control R1	Wounded R3
5	Pooled sample	–	Control R2

R = Replications of the treatments.

**Figure 2:**
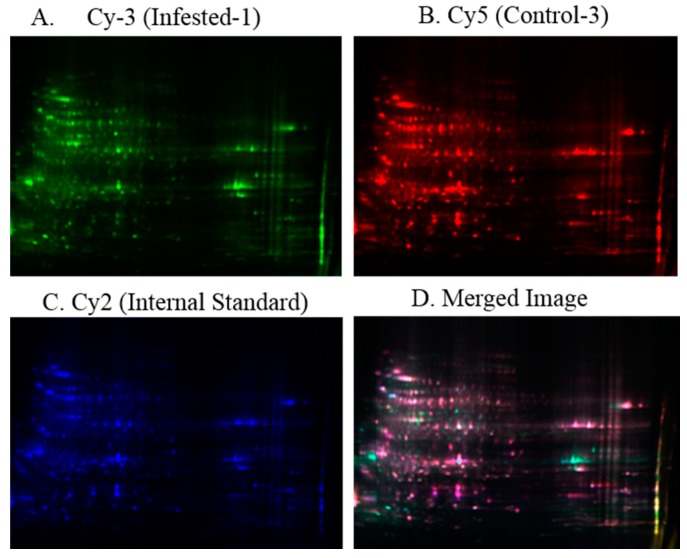
2D-DIGE images of date palm proteins. The protein sample of Infested-1, Control-3 and internal standard (pooled of all the samples) are individually labeled with Cy dyes, mixed together and separated by 2D-DIGE followed by image scanning. (**A**) Image of date palm infested sample and labeled with Cy3 dye; (**B**) Image of date palm control sample labeled with Cy5 dye; (**C**) Image of date palm sample pooled from all and labeled with Cy2 dye; and (**D**) Overlay gel of infested, and control samples along with internal standard.

After labeling with Cy dyes, two samples were mixed with different combinations along with internal standard and electrophoresed on the same gel except one gel contained a single sample with internal standard. The representative gels of 2D-DIGE after scanning with a fluorescence gel scanner, Typhoon imager (Trio) (GE Healthcare), are shown in Figure 2.

Progenesis Samespots software version 3.3 (Nonlinear Dynamics Ltd., Newcastle, United Kingdom) was used to statistically analyze the protein expression among control, infested, and wounded samples.

**Figure 3:**
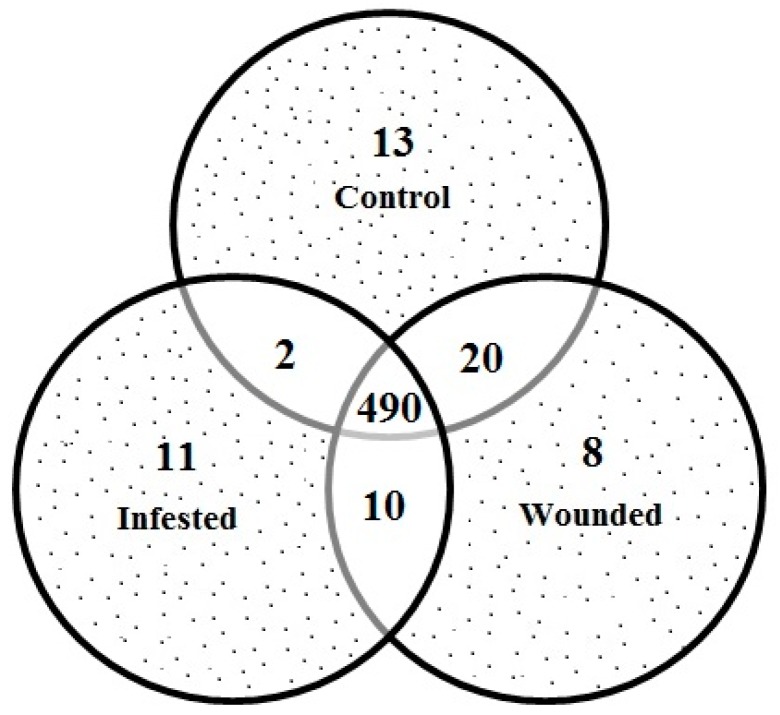
Venn diagram for the relative distribution of differentially expressed proteins spots in control, mechanically wounded and RPW infested date palm samples. The non-overlapping segments of diagram represent the number of proteins that were significantly upregulated (>1.5-fold) in the corresponding group when compared with the other two groups. The overlapping region between any two groups represents the number of proteins spots significantly upregulated (>1.5-fold) compared to the third one. While the central overlapping region represents the protein spots where no any statistically significant change was observed.

A total of 522 well-resolved protein spots were observed on each gel, and out of them, 32 spots showed statistically significant differences (*p* ≤ 0.05, and intensity fold change ≥ 1.5) among expressions of proteins in either of this combination. Among 32 differential expressed spots, 11 were upregulated in infested and eight in wounded. However, there was downregulation of 13 spots in both infested and wounded when compared with control (Figure 3).

Interestingly, spots from the wounded samples showed the same trend of up or downregulation compared to control, but fold change intensity is lower than the infested samples. Differential protein patterns were also observed in the infested and wounded samples that are of our interest. The same spot (protein) was upregulated in the infested and downregulated in the wounded compared to control and *vice versa*. A majority of the upregulated spots (spot numbers 414, 243,554, 300, 447, 994, 1281, 1073 and 438) followed a unique trend of upregulation like (I > W > C) except two spots (1308 and 1287) where the pattern was observed like (I > C > W). Similar to upregulation, almost the same trend was observed among majority of downregulated spots in reverse order C > W > I (spots numbers 1027, 987,976, 781, 798, 1023, 951, 1020, 683, 1086, 1163 and 884). However, some deviations were noticed in some spots (221, 235, 234, 506, 822 and 318) where the expression pattern was observed as W > C > I, except in two spots (137 and 313) this pattern was like W > I > C. Our data clearly demonstrates that RPW infestation relatively enhances the modulation of proteins upregulated or downregulated with few minor variations. The relative expression patterns are quite intriguing and previous reports also suggest such trends in plants [34,35].

### 2.2. Protein Identification by Mass Spectrometry

Proteomic methodologies for differential expression are quite tedious by nature; however, it provides highly reliable information once a modulated peptide has been identified. Final identification subsequent to proteomic methodology involves identification procedures involving mass spectrometric analyses. A state of the art proteomic methodology MALDI-TOF used for the identification of proteins by peptide mass fingerprinting was employed to characterize peptides modulated in date palm stems subsequent to infestation with RPW. The above-described 32 differentially expressed spots were ex*cis*ed from preparative gels, digested enzymatically with trypsin, and subjected to mass spectrometry MS analysis. Data were examined using BioTools 3.2 (Bruker Daltonics, Bremen, Germany) in combination with the Mascot search algorithm (version 2.0. 04) against green plants database. MS results showed that identified 21 spots matched with previously reported proteins found in databases (Table 2). We were unable to characterize 11 protein spots as there was no protein matching proteins in the database. It is quite possible that date palm plant has some unique proteins not found in other plants. Once the date palm genome is deciphered, these unique proteins will be of much interest. In protein identification a small number of different spots showed the same identified proteins, indicating the presence of isoforms of a specific gene or these emerged because of post-translational modifications (PTMs) differences in molecular weight and pI. In our case V-type proton ATPase catalytic subunit A (spot numbers 221 and 243), and probable glycerol-3-phosphate acyltransferase 8 (spot numbers 683 and 781) belong to these categories.

The identities of differentially expressed proteins were confirmed by their comparison with the previously characterized proteins in Swiss-Prot database. Among these 33% were characterized based on their homology with *Arabidopsis thaliana* proteins, 14.2% *Oryza sativa* (*subspecies Japonica*) (rice), and 9.5% *Sorghum bicolor* (Sorghum), respectively. Only 4.7% proteins were confirmed from *Zea* *mays*, *Solanum lycopesicum*, *Eucalyptus gunnii* (Cider gum), *Hevea brasiliensis* (Para rubber tree), *Citrus unshiu* (Satsuma mandarin), *Carica papaya* (papaya), *Medicago truncatula* (Barrel medic), *Spinacia oleracea* (Spinach), and *Hordeum vulgare* (Barley) proteome.

Moreover, differentially expressed proteins had been characterized as ion transport (6), lipid biosynthesis (2), protein folding (1), plant defense (1), ethylene signaling pathway (1), carbohydrate metabolism (1), proteolysis (1), *S*-adenosylmethionine biosynthetic process (1), nitrate assimilation (1), porphyrin biosynthesis (1), transcription (1), lignin biosynthesis (1), cytoskeletal protein (1), calcium ion binding (1) and unknown (1).

It is worth mentioning that among the 21 proteins identified through homology comparison, six belong to the ion transport family of proteins that are stress related, while several others could potentially be involved in response to RPW infestation. These were V-type proton ATPase catalytic subunit A (*Zea mays* (Maize) (spot numbers. 221, 243). The spot 221 were suppressed 1.5-fold in the infested samples relative to control. However, spot 243 was much higher in infested samples (5.3-fold) compared to control. V-type proton ATPase is a proton pump present inside plant vacuoles which plays an important role in plant salinity tolerance [36,37]. V-type proton ATPase plays a crucial role in maintenance of ion homeostasis inside plant cells by acidifying compartments of the vacuole [38,39]. It maintains electrochemical H^+^-gradient to drive the transport of Na^+^ into the vacuole lumen, compartmentalizing this toxic ion from the cytoplasm and maintaining low cytoplasmic Na^+^ concentrations. Upregulation of this enzyme was also reported in response to stress in other plants as well [40,41,42,43]. The upregulation of enzyme V-type proton ATPase might provide insights into understanding the infestation of date palm and could be a useful marker for early diagnosis of RPW. Another spot (spot number 414) identified as a differential protein and upregulated in infested samples corresponded to ATP synthase subunit β, mitochondrial.

**Table 2:** Differentially expressed proteins between control, wounded and infested date palm stem by MALDI-TOF peptide mass fingerprinting after 2D-DIGE.

Spot Number	FC (I/C)	FC (W/C)	Accession (Uniprot)	Protein Description	Function	pI	*M*_W_	Coverage	Score	Organism
1308	1.60↑	3.03↓	P27161	Calmodulin	M: Calcium ion binding	4.18	16,950	69	229	*Solanum lycopersicum* (Tomato)
781	2.42↓	1.67↓	Q5XF03	Probable glycerol-3-phosphate acyltransferase 8	Lipid biosynthesis	9.14	56,345	25	60	*Arabidopsis thaliana* (Mouse-ear cress)
221	1.50↓	1.17↑	P49087	V-type proton ATPase catalytic subunit A	Ion transport	5.89	62,198	25	62	*Zea mays* (Maize)
798	2.08↓	1.48↓	O04854	Caffeoyl-CoA *O*-methyltransferase	Lignin biosynthesis	5.02	28,010	12	72	*Eucalyptus gunnii* (Cider gum)
414	2.77↑	1.28↑	P29685	ATP synthase subunit β, mitochondrial	ATP hydrolysis coupled proton transport	5.98	60,335	35	75	*Hevea brasiliensis* (Para rubber tree)
994	1.68↑	1.56↑	Q94FT8	Non-symbiotic hemoglobin 3	Iron and oxygen transport	9.83	18,614	65	65	*Oryza sativa* subsp. japonica (Rice)
1281	1.76↑	1.58↑	Q94FT8	Non-symbiotic hemoglobin 3	Iron and oxygen transport	9.83	18,614	83	62	*Oryza sativa* subsp. japonica (Rice)
137	1.66↑	1.99↑	P22953	Heat shock 70 kDa protein 1	Stress response, plant defense,	5.03	71,712	38	88	*Arabidopsis thaliana* (Mouse-ear cress))
1073	2.07↑	1.50↑	Q9SVQ0	Ethylene-responsive transcription factor ERF062	Ethylene signaling pathway	9.46	44,283	37	68	*Arabidopsis thaliana* (Mouse-ear cress)
243	5.31↑	1.77↑	Q9SM09	V-type proton ATPase catalytic subunit A	Hydrogen ion transport	5.29	68,923	34	70	*Citrus unshiu* (Satsuma mandarin)
554	1.99↑	1.39↑	Q1PFG1	F-box protein At1g66490	Uncharacterized	8.75	43,577	26	65	*Arabidopsis thaliana* (Mouse-ear cress)
300	2.98↑	2.13↑	Q39251	Actin-depolymerizing factor 2	Cytoskeletal protein	5.24	15,963	36	65	*Arabidopsis thaliana* (Mouse-ear cress)
447	2.36↑	1.17↑	B1A986	NAD(P)H-quinone oxidoreductase subunit 4L, chloroplastic	Oxidoreductaseactivity, ATP synthesis coupled electron transport	9.65	11,327	54	67	*Carica papaya* (*papaya*)
1020	2.81↓	2.35↓	Q9SKQ0	Peptidyl-prolyl *cis*-*trans* isomerase CYP19-2	B: protein folding	8.33	1868	48	63	*Arabidopsis thaliana* (Mouse-ear cress)
683	2.58↓	1.69↓	Q5XF03	Probable glycerol-3-phosphate acyltransferase 8	Lipid biosynthesis	9.14	56,345	29	60	*Arabidopsis thaliana* (Mouse-ear cress)
506	1.36↓	1.65↑	Q8W0A1	β-galactosidase 2	Carbohydrate metabolic process	5.59	92,630	19	62	*Oryza sativa* subsp. japonica (Rice)
822	1.92↓	1.06↑	C5X3M7	Putative uncharacterized protein Sb02g009233	Nucleic acid binding	5.60	14,533	88	73	*Sorghum bicolor* (Sorghum)
318	1.48↓	1.62↑	A5C9D0	Putative uncharacterized protein	Proteolysis	9.41	119,434	20	71	*Sorghum bicolor* (Sorghum)
438	2.24↑	1.02↑	A4PU48	S-adenosylmethionine synthase	Adenosylmethionine biosynthesis	5.59	43,708	29	63	*Medicago truncatula* (Barrel medic)
884	1.61↓	1.45↓	P23312	S Nitrate reductase [NADH]	Nitrite assimilation	6.25	104,703	68	62	*Spinacia oleracea* (Spinach)
313	2.44↑	2.47↑	Q42836	Delta-aminolevulinic acid dehydratase, chloroplastic)	Porphyrin biosynthesis	6.05	46,639	46	80	*Hordeum vulgare* (Barley)

Arrows indicate the proteins up (↑) and down (↓) regulations, FC = Fold change, I = RPW Infested samples, W = Mechanically Wounded samples; pI = Isoelectric point, *M*_W_ = Molecular Weight.

The expression of calcium binding protein, calmodulin (spot no. 1308) increased (1.6-fold) in response to the infestation of RPW. Calmodulin is a calcium-binding messenger protein and regulates downstream functions in response to Ca^2+^. It has several targets including ion channel and a large number of enzymes (i.e., kinases, phosphatases, cytoskeletal proteins, synaptic proteins, cell cycle proteins) [44,45,46,47] and also is involved in heat-shock signal transduction [48]. RPW infestation associated upregulation of this protein probably is associated with enhanced signaling activity associated with infestation. The signaling cascade involves a number of proteins, and its interactions with several above-described proteins have been previously reported.

Protein belonging to S-adenosylmethionine synthase (spot number 438) showed the increased expression (2.2-fold) in infested sample relative to control. This enzyme catalyzes the synthesis of *S*-adenosylmethionine from methionine and ATP. Also, expression was upregulated by 2.4-fold in spot number 313, homologous to Delta-aminolevulinic acid dehydratase, chloroplastic) as compared to control. This enzyme is involved in the formation of porphobilinogen [49]. The expression of the spot number 137, homologous to heat shock 70 KDa proteins (HSP 70), was increased (1.66-fold) in the infested samples as compared to control. However, its expression was increased more in the wounded (~2-fold) relative to control. HSP 70 protein is a chaperone that assists the folding process of newly synthesized proteins and minimizes aggregation [50]. High expression of this protein under stress conditions has also been reported previously [50,51]. Several other HSPs have also been reported in response to different stress conditions like because of pea and *Erysiphe pisi* interaction [52], pea and *Mycosphaerella pinodes* interaction [53], or triticale under low N fertilization level [54]. The over-expression of these stress responsive proteins should not be surprising, because it is a natural defense mechanism that responds, as mentioned above, to any outer biotic/abiotic stress. The over expression of HSP 70 in present study could be a useful marker against RPW infestation.

The 2-spots (781 and 683) matching glycerol-3-phosphate acyltransferase, an essential enzyme for glycerolipid biosynthesis, were found to be down regulated in infested samples. Another down regulated spot (506) matched with protein β-galactosidase 2 was identified. This enzyme is responsible for non-reducing β-d-galactose residues in β-d-galactosides.

Furthermore, spot number 1073 was identified as ethylene response factor (ERF1) and its expression was upregulated (2.07-fold) in infested date palm sample relative to control. ERF1 is a member of the novel family of plant-specific transcriptional factors in *Arabidopsis thaliana* [55], and it is activated by either ethylene (ET) or jasmonate (JA) and is activated synergistically by both hormones [56]. ERF1 regulates defense response genes to the necrotrophic fungi *B. cinerea* and *P. cucumerina* by integrating ET and JA defense responses in *Arabidopsis* [57,58]. ERFs have been reported to affect a number of developmental processes, and are also differentially adapted to biotic or abiotic stresses such as pathogen attack, wounding, extreme temperature, and drought [59,60]. Our results suggest that ERF1 could be a key element in defense against RPW attack.

Another spot 884 matching with nitrate reductase was suppressed (1.6-fold) in date palm infested sample compared to control. This enzyme plays a key role in the synthesis of nitric oxide (NO) [61], an important signaling molecule mediating physiological and developmental processes. Also, NO plays an important role in plant responses to biotic and abiotic stresses [62,63,64]. Furthermore, NO was also reported to modulates ethylene, salicylic acid, and jasmonic acid-signaling pathways and abscisic acid (ABA)-induced stomatal closure [65,66].

Another important protein identified as nonsymbiotic hemoglobins 3 (spot numbers 994 and 1281) was upregulated in infested and wounded samples that are induced in plants during hypoxic stress. Nonsymbiotic hemoglobin AHb1 plays an important role in NO detoxification in *Arabidopsis* by scavenging NO and reducing its emission under hypoxic stress. These proteins were reported to protect plants during hypoxia or other similar stresses [67,68] and scavenge NO produced in stress conditions [69]. Nonsymbiotic hemoglobin AHb1 plays an important role in NO detoxification in *Arabidopsis thaliana* by scavenging NO and reduces NO emission under hypoxic stress [70].

A significant increase (2.98-fold) of actin depolymerizing factors (ADFs) (spot number 300) was found in infested samples compared to control. ADF is a small actin-binding protein and involved in plant growth, development stress response, and pathogen defense [71,72,73]. The role of actin had also been reported in response to plant hormones and biotic or abiotic stresses [71,74]. The ADFs have been found related to plant resistance to various pathogens in *Arabidopsis* and barley [75,76]. The energy produced by the depolymerization and polymerization of actin is used for the directional movement of cells, which is necessary for wound healing, immune response, embryonic development and development of tissues [77,78,79]. In this process a number of actin binding proteins, such as profillin, actin depolymerizing factor (ADF)/cofilin, myosin, fibrin and villin, are also involved.

Spot number 447 corresponding to NAD(P)H-quinone oxidoreductase subunit 4L, chloroplastic showed a significant increase (2.34-fold) in infested date palm samples as compared to control. NAD(P)H-quinone oxidoreductase is an important enzyme that is involved in the detoxification of quinones and their derivatives [80,81]. Taken together, this enzyme increased to reduce oxidative stress and to detoxify toxic molecules produced by the stress.

The spot no. 1020 corresponding to peptidyl-prolyl *cis*-*trans* isomerase (PPIase) CYP19-2 was downregulated (2.8-fold) in the infested samples. This enzyme catalyses the *cis*-*trans* isomerisation process of proline residues during protein folding [82]. Important groups of proteins, such as cyclophilin proteins, have this PPIase domain. Cyclophilins have been shown to be involved in a wide range of cellular processes like stress tolerance [83], cell division [84], transcriptional regulation [85], protein trafficking, [86] cell signaling [87], pre-mRNA splicing [88], and molecular chaperoning [89]. Taken together, a majority of identified proteins were directly or indirectly related to defense of date palm and could be exploited for the early diagnosis of RPW infestation. The information obtained here must contribute to clarification of plant defensive mechanism and RPW management.

Our current study also reveals that 21 differentially expressed peptides identified through our proteomic analyses have a major group of ion transport proteins. Furthermore, proteins belonging to various groups like lipid biosynthesis, protein folding, plant defense, carbohydrate metabolism, lignin biosynthesis, cytoskeletal proteins and a few others in plant stem, manifest integrated signaling mechanisms involved in injury/infestation processes. Once the date palm genome is revealed completely, study of proteomics networks involved upon infestation or injury will be of great interest. If we compare our current findings with the previous proteomic optimization from plant leaves [29]), it is clear that stem and leaves have different metabolic activities. Particularly, the stem part of the date palm plant serves several purposes such as storage, transport to aerial parts and maintaining overall plant integrity. Our report on differential proteomics of leaves showed 11 modulated peptides falling into only three categories, *i.e.* stress/defense, photosynthetic and ion transport. These findings make more sense as the aerial part of this plant is involved with photosynthetic processes whereas stem mainly contributes to plant overall structure and defense. The 11 peptides having no homologous counterparts in the plant proteome will be part of our future research interests. Diversity of modulated proteins either upon artificial injury or red palm weevil infestation shows integrated signaling in various metabolic aspects once the plant is exposed to unnatural circumstances either through injury or infestations.

## 3. Experimental Section

### 3.1. Date Palm Material and Infestation with RPW Larvae

Six-year old tissue cultured date palm plants of Khudry cultivar were obtained from Al Rajhi Tissue Culture Laboratory, Riyadh, Saudi Arabia*.* These plants were divided into 3 groups, with three replicates each, and were then used for mechanical wounding and infestation with RPW as described previously [90]. Group one was artificially infested (I) with RPW larvae, group two artificially wounded (W), and the third group was kept without any treatment as control (C). For artificial infestation each plant was introduced with 5 second instar RPW larvae by making tiny holes in the stem using drill machine with 6-mm size bit to accommodate larva. After treatment the stem part of the plants were wrapped up with fine steel mesh. The stem samples were taken after 3-days of infestation and stored at −80°C until use.

### 3.2. Protein Extraction and SDS-PAGE

Total proteins from control, infested, and wounded date palm stem samples (three replicates from each sample) were extracted using phenol/SDS extraction method as described by Gomez-Vidal *et al*, [33] with minor modifications [29]. Briefly, stem tissues (one gram) were ground into fine powder in liquid nitrogen in pestle/mortar and suspended in 5 mL phenol and 5 mL dense SDS buffer (30% *w*/*v* sucrose, 2% *w*/*v* SDS, 0.1 M Tris-HCl, pH 8.0, 5% *v*/*v* 2-mercaptoethanol). After mixing and vortexing, mixture was centrifuged for 5 min at 10,000 rpm at 4 °C. The upper phenolic phase was collected and precipitated with five volumes of cold 0.1 M methanolic ammonium acetate. After incubating at −20 °C for 30 min, precipitated proteins were recovered by centrifugation at 1000 rpm for 5 min at 4 °C and then washed two times with cold methanol solution containing 0.1 M ammonium acetate and then two times with cold 80% *v*/*v* acetone. The protein pellet was recovered each time by centrifugation at 8000 rpm for 5 min. The final protein pellet was air-dried at room temperature and suspended in 100 mM Tris buffer pH 8.0 and the then added equal volume of 2X SDS-reducing buffer (100 mM Tris–Cl (pH 6.8), 4% SDS, 0.2% bromophenol blue, 20% glycerol) containing 200 mM mercaptoethanol. SDS-PAGE analysis was carried out as described by Laemmli [91]. The gel was stained with Coomassie brilliant blue G-250 with constant and gentle agitation overnight. After staining the gel was placed in destining solution until the background becomes clear.

### 3.3. Two-Dimensional Difference Gel Electrophoresis (2D-DIGE)

The dried protein sample for 2D-DIGE was solubilized in rehydration buffer containing the chaotropic agent urea, alongside surfactants 3-[(3-cholamidopropyl)dimethylammonio]-1-propanesulfonate (CHAPS) and thiourea (7 M urea, 2 M thiourea, 2% CHAPS *w*/*v*, 2% Dithiothreitol (DTT), 0.5% Immobilized pH Gradient (IPG) buffer pH 3–11, 0.002% bromophenol blue) by shaking at 150 rpm for 1 h at 25 °C. The insoluble residue was removed by centrifugation at 12,000 rpm for 10 min. Protein concentration was measured using 2-D Quant kit (GE Healthcare, Buckinghamshire, United Kingdom) in accordance with to the manufacturer’s protocol and using bovine serum albumin (BSA) as a reference standard. The samples were further cleaned for 2D using the 2D Clean-Up Kit (GE Healthcare, Buckinghamshire, United Kingdom) and solubilized in buffer (7 M urea, 2 M thiourea, 2% CHAPS, 30 mM Tris-Cl, pH 8.5) without DTT and IPG buffer, and then they were quantified again using 2-D Quant kit (GE healthcare, Buckinghamshire, United Kingdom). Protein was aliquoted to required amount (300 µg) and frozen.

After adjusting the pH to 8.5, each protein sample was labeled with CyDye Fluor minimal dyes (GE healthcare, Buckinghamshire, United Kingdom) according to manufacturer’s recommendation. Briefly, 50 µg each protein sample was incubated with 400 pmol CyDye Fluor minimal dyes on ice for 30 min in the dark. The control, wounded, and infested samples were labeled alternatively with Cy3 or Cy5 (Table 1). Internal standard containing equal amount of proteins from each sample was labeled with Cy2. The reaction was stopped by adding 1 µL of 10mM lysine solution and incubated 10 min on ice and proteins samples were combined according to experimental design as shown in Table 1.

For 2D-DIGE, five IPG Immobiline DryStrips 24 cm pH 3-11 (GE Healthcare, Buckinghamshire, United Kingdom) were rehydrated for 16 h with rehydration buffer (7 M urea, 2 M thiourea, 2% CHAPS *w*/*v*, 0.2% DTT, 0.5% IPG buffer pH 3–11, 0.002% bromophenol blue) containing the protein samples for each gel. Isoelectric focusing was performed with Ettan IPGphor3 IEF unit (GE Healthcare, Buckinghamshire, United Kingdom) at 50 µA per strip at 20 °C according to following programme:1) 500V for 1 h, 2) 1000V for 1 h, 3) 8000V for 3 h, 4) 8000 V for 45,000Vh. Strips were immediately equilibrated after Isoelectric focusing for 15 min in equilibration buffer 1 (50 mM Tris-HCl, pH 8.8; 6 M urea; 20% (*v*/*v*) glycerol and 2% (*w*/*v*) SDS) containing 2% DTT at room temperature under gentle agitation, and then by equilibration buffer 2 (50 mM TrisHCl, pH 8.8; 6 M urea; 20% (*v*/*v*) glycerol and 2% (*w*/*v*) SDS) containing 2.5% iodoacetamide. Second-dimension sodium dodecyl sulfate polyacrylamide gel electrophoresis (SDS-PAGE) was performed on 5%–20% polyacrylamide gradient gels using the Ettan DALT six vertical unit (GE Healthcare, Little Chalfont, UK) at 15 °C for 1W per gel for 1 h and then 2W per gel until the bromophenol blue dye reached the end of gel. Polyacrylamide gradient gels were prepared on low fluorescence glass using 2D Optimizer (Nextgen Sciences).

### 3.4. Image Acquisition and Analysis

The 2D gels were scanned using fluorescence gel scanner, Typhoon imager (Trio) (GE Healthcare, Uppsala, Sweden), using appropriate wavelengths and filters for Cy2, Cy3 and Cy5 dyes according to manufacturer’s recommended protocol. The 2D gels images were analyzed using Progenesis Same Spot software version 3.3 (Nonlinear Dynamics Ltd, Newcastle upon Tyne, UK). The differential expression was ascertained using normalized protein spots in the Cy5 and Cy3 channels compared to the internal standard (Cy2). The spots of infested and wounded date palm samples were compared to control samples. One-way ANOVA was used to calculate the fold difference values and *p*-values. A threshold level was set at 1.5-fold up or downregulation, at *p* ≤ 0.05 level.

### 3.5. Protein Identification by Mass Spectrometry

Preparative 2D gels were run using 700 µg total protein sample obtained by pooling all the samples present in the experimental design. The gels were fixed in ethanol (35% *v*/*v*), with phosphoric acid (2% *v*/*v*) overnight and then washed three times with water for 30 min each time. Then the gels were incubated for 1 h in methanol (34% *v*/*v*) containing ammonium sulfate (17% *w*/*v*) and phosphoric acid (3% *v*/*v*) for I h and after that 0.5% g/L Coomassie G-250 were added. The gels were stained for 5 days followed by rinsing in Milli Q water and stored until spots were picked and identified by Mass spectrometry (UltraFlexTrem, Bruker Daltonics, Bremen, Germany). Corresponding differential spots were matched to a colloidal Coomassie-stained image of the preparative gel, which was first mapped to the reference image.

The differential protein spots were manually excised form Coomassie-stained preparative gels and washed with solution containing 50 mM Ammonium bicarbonate. Then, the gel pieces were destained with 50 mM ammonium bicarbonate and 50% acetonitrile followed by 100% acetonitrile. Once destained, gel pieces were dried using vacuum centrifugation at 40 °C for 5 min. Dried gel pieces were rehydrated and digested with 10 µL trypsin at a concentration of 2ng/µL (Promega, Madison, WI, USA) in 25 mM NH_4_HCO_3_ pH 8.0 at 4 °C for 60 min, and digestion was continued for an additional 16–24 h at 37 °C. The digestion was stopped and digest mixtures were transferred to 0.5 mL tube, and the peptides were extracted by adding 50% acetonitrile having 0.1% Trifluoroacetic acid followed by drying to 10 µL using vacuum centrifugation. The 0.5 µL peptides was mixed with the matrix (10 mg α-Cyano-4-hydroxycinnamic acid in 1 mL of 30% acetonitrile containing 0.1% TFA) and applied on MALDI-target and dried before MS analysis, and then it was subjected to MALDI-TOF-MS (UltraFlexTrem, Bruker Daltonics, Bremen, Germany) in a positive mode. Peptide mass fingerprints were processed using flex analysis software (version 2.4, Bruker Daltonics, Bremen, Germany). MS data were interpreted by BioTools 3.2 (Bruker Daltonics, Bremen, Germany) in combination with the Mascot search algorithm (version 2.0. 04) against Swiss-Prot database for green plants. The validity/accuracy of identified proteins was only accepted when the mascot score was ≥ 60.

## 4. Conclusions

Date palm infestation with RPW is a key threat to the survival of date palm trees in the Kingdom of Saudi Arabia and other Gulf countries. We have utilized proteomic methodologies to identify molecular changes associated with RPW infestation of this plant. Though our interest is to identify RPW-specific molecular responses, it was however interesting to note that similar molecular moieties are upregulated in artificial wounding as well as RPW infestation. Nevertheless, relative modulation (downregulation/upregulation) remained quite differential. As both mechanical injury and RPW infestation impinges upon the same molecular moieties of date palm stem, we would have to establish certain baselines of proteomic changes for characterizing RPW-specific molecular changes. This study mainly provides a proof of concept that the stem, the hard part of the date palm tree, is amenable to molecular analytical procedures for understanding infestation with RPW. Furthermore, this work opens new avenues for understanding the proteome of this important tree. Techniques established and data generated will be crucial for date palm scientists in understanding diseases/infestation associated with the physiological process in this plant, in addition to developing new cultivars.
